# Accuracy of Xpert *Clostridium difficile* assay for the diagnosis of *Clostridium difficile* infection: A meta analysis

**DOI:** 10.1371/journal.pone.0185891

**Published:** 2017-10-09

**Authors:** Yuanyuan Bai, Xiaorong Sun, Yan Jin, Yueling Wang, Juan Li

**Affiliations:** 1 Department of Clinical Laboratory, Shandong Provincial Hospital Affiliated to Shandong University, Jinan, PR China; 2 Department of Pathology, Jinan Women and Children’s Health Hospital, Jinan, PR China; Cornell University, UNITED STATES

## Abstract

**Background:**

There is an urgent need for rapid and accurate microbiological diagnostic assay for detection of *Clostridium difficile* infection (CDI). We assessed the diagnostic accuracy of the Xpert *Clostridium difficile* assay (Xpert CD) for the diagnosis of CDI.

**Methods:**

We searched PubMed, EMBASE, and Cochrane Library databases to identify studies according to predetermined criteria. STATA 13.0 software was used to analyze the tests for sensitivity, specificity, positive likelihood ratio, negative likelihood ratio, diagnostic odds ratio, and area under the summary receiver operating characteristic curves (AUC). QUADAS-2 was used to assess the quality of included studies with RevMan 5.2. Heterogeneity in accuracy measures was tested with Spearman correlation coefficient and *Chi*-square.

**Results:**

A total of 22 studies were included in the meta-analysis. The pooled sensitivity (95% confidence intervals [CI]) was 0.97 (0.95–0.99) and specificity was 0.95 (0.94–0.96). The AUC was 0.99 (0.97–0.99). Significant heterogeneity was observed when we pooled most of the accuracy measures of selected studies.

**Conclusions:**

The Xpert CD assay is a useful diagnostic tool with high sensitivity and specificity in diagnosing toxigenic CDI, and this method has excellent usability due to its rapidity and simplicity.

## Introduction

Over the last decades, *Clostridium*. *difficile* infection (CDI) has emerged as a leading cause of nosocomial diarrhea, accounting for 15% to 25% of antibiotic-associated diarrhea [1]. CDI is a life-threatening and costly disease, associated with a one-month mortality ranging from 3 to 30% [2] and more than $1.5 billion in costs a year in the United States [3]. The main virulence factors of *C*. *difficile* are toxins A and B, which are respectively encoded by the *tcdA* and *tcdB* genes, with expression of either toxin sufficient to cause disease [4]. Furthermore, approximately 6 to 12.5% toxigenic strains also produce a third toxin known as binary toxin, encoded by the *cdt* locus [5]. Although its role in CDI pathogenesis has been unclear, the presence of binary toxin in combination with a single-nucleotide deletion at base pair 117 within the negative toxin regulator gene *tcdC* is considered a hallmark for ‘hypervirulent’ 027/NAP1/BI (PCR-ribotype 027 or NAP1 according to pulse-field gel electrophoresis typing, or BI according to restriction enzyme analysis typing) strains which have caused several important outbreaks of severe CDIs [6]. These strains have been shown to produce a large amount of toxins *in vitro* and are associated with erythromycin and newer fluoroquinolones resistance.

The diagnosis of CDI is usually made based on the combination of clinical presentation and laboratory tests. Despite numerous laboratory methods are now available, the diagnosis of CDI still remains a challenge. The anaerobic toxigenic culture (TC) and culture cytotoxicity neutralization assay (CCNA) were often used as the laboratory reference tests for detecting *C*. *difficile*. However, the two tests have limitations such as a long turnaround time (48–72 hours) and technical complexities [7], which may result in delayed proper treatment. In practice, enzyme immunoassays (EIAs) for detecting *C*. *difficile* toxins have been the most frequently employed tests in clinical labs. There are a number of commercially available EIAs for *C*. *difficile* toxins, which are used conveniently and provide a quick result for a low cost with good specificity. However, it was ultimately demonstrated that EIAs cannot be used as standalone tests due to its low sensitivity [8]. Therefore, accurate and rapid diagnosis of CDI is essential for proper treatment and infection control.

The technological advancement of molecular biotechnologies has been of interest for detecting CDI. Recently, Nucleic acid amplification tests (NAATs) for the direct detection of toxigenic *C*. *difficile* have been developed and implemented in many labs due to its high sensitivity as good as TC. Currently, several NAATs have been cleared by Food and Drug Administration (FDA) [9] and supported by recent guidelines by the American Society of Microbiology [10]. Most commercially available NAATs target the *tcdB* gene, which is produced by all the toxigenic strains of *C*. *difficile* [11]. The Xpert CD assay (Cepheid, Sunnyvale, CA, USA) is a multiplex PCR assay. As described in detail previously by Burnham, C. A. D et al [9], the unique features of this assay are that it not only detects *tcdB* but also the binary toxin genes and the deletion at nucleotide 117 on *tcdC* (Δ117) as hallmarkers for presumptive identification of ‘hypervirulent’ 027/NAP1/BI strains. This assay is among the simplest to perform and is also the most rapid of the available NAATs that the turn-around time is about 1 hour.

According to the Society for Healthcare Epidemiology of America and the Infectious Diseases Society of America guidelines, “…PCR testing appears to be rapid, sensitive, and specific and may ultimately address testing concerns. More data on utility are necessary before this methodology can be recommended for routine testing”. Several previous studies have examined the performance of the Xpert CD assay for detecting CDI, however, the sensitivity and specificity results have been inconsistent. In the present study, a new meta-analysis was performed to comprehensively evaluate the overall diagnostic accuracy of the Xpert CD assay in detecting CDI compared with reference tests.

## Methods

We followed the Preferred Reporting Items for Systematic Reviews and Meta-Analyses (PRISMA) guidelines in our study.

### Literature search

Original articles published in English up to the end of July 2017 were searched in PubMed, EMBASE and Cochrane Library databases by two investigators (Y. Bai and J. Li). The search terms used were as follows: *Clostridium difficile* AND (Xpert *C*. *difficile* OR molecular diagnostic techniques). Reference lists from included studies were also searched.

### Study criteria

We systematically searched the literature using the following predetermined inclusion criteria. Studies evaluating Xpert CD as a diagnostic test for CDI were eligible for inclusion if the studies 1) described original research; 2) performed stool samples analyses from human patients, either children or adults; 3) compared Xpert CD to a reference method–either CCNA or anaerobic TC; 4) had extractable data to fill the 4 cells of a 2 × 2 table for diagnostic tests (true positives (TP), true negatives (TN), false positives (FP), and false negatives (FN)).

Relevant publications were excluded if they were duplicated articles, letters without original data, animal studies, case reports, editorials, and reviews. Studies with fewer than 20 samples were also excluded to reduce selection bias. Articles that contain data from infants were excluded because infants rarely develop clinical infection.

### Data extraction

Two investigators (Y. Bai and J. Li) extracted data from full text of the included studies independently. Disagreements were resolved by consensus. Information was extracted on the first author, publication year, country where the study was conducted, sample size, reference tests the diagnosis used, the number of TP, the number of FP, the number of FN, and the number of TN. These were summarized as sensitivity, TP/(TP+FN); specificity, TN/(TN + FP);and prevalence, (TP+FN)/(TP+FN+TN+FN).

### Quality of study reports

We applied the Quality Assessment of Diagnostic Accuracy Studies (QUADAS-2) to assess the quality of included studies (http://www.bris.ac.uk/quadas/), an updated version of the original software [12]. QUADAS-2 is used in systematic reviews to evaluate the risk of bias and applicability of diagnostic accuracy studies, and consists of four key domains: patient selection, index test, reference standard, and flow and timing. Each domain is assessed for risk of bias and the first three are also evaluated for applicability. Signaling questions were included to assist in judgments about the risk of bias [13]. If the answers to all signaling questions for a domain were “yes,” the risk of bias is judged as “low;” if any signaling question in a domain was “no,” risk of bias is judged as “high.” The unclear bias should only be used if insufficient information was supplied [13]. Applicability was judged as low, high, or unclear with the similar criteria.

### Statistical analysis

#### Accuracy estimates

Meta-analyses were performed using two software programs: STATA 13.0 (Stata Corporation, Texas, USA) and Cochrane RevMan 5.2. Sensitivity, specificity, positive likelihood ratio (PLR), negative likelihood ratio (NLR), and diagnostic odds ratio (DOR), forest plots and summary receiver operating characteristic (SROC) curves were analyzed with the ‘midas’ module for STATA 13.0, based on the random model effect. Quality of studies was assessed with RevMan 5.2.

#### Heterogeneity

We used chi-square test and *I*^*2*^ (*p* < 0.05 and *I*^*2*^ > 50% indicated significant heterogeneity) to identify heterogeneity. The methods to evaluate the heterogeneity were described detailedly in our previous published study [14]. The further reasons for heterogeneity of the data were addressed by performing subgroup analyses on prespecified variable: the calculated prevalence of *C*. *difficile* (<15% and ≥15%) and the sample size (< median size 246 and ≥246).

#### Fagan's nomogram

The method to depict visual Fagan's nomogram was described in detail as previously [15].

## Results

### Characteristics of selected studies

**Fig 1:**
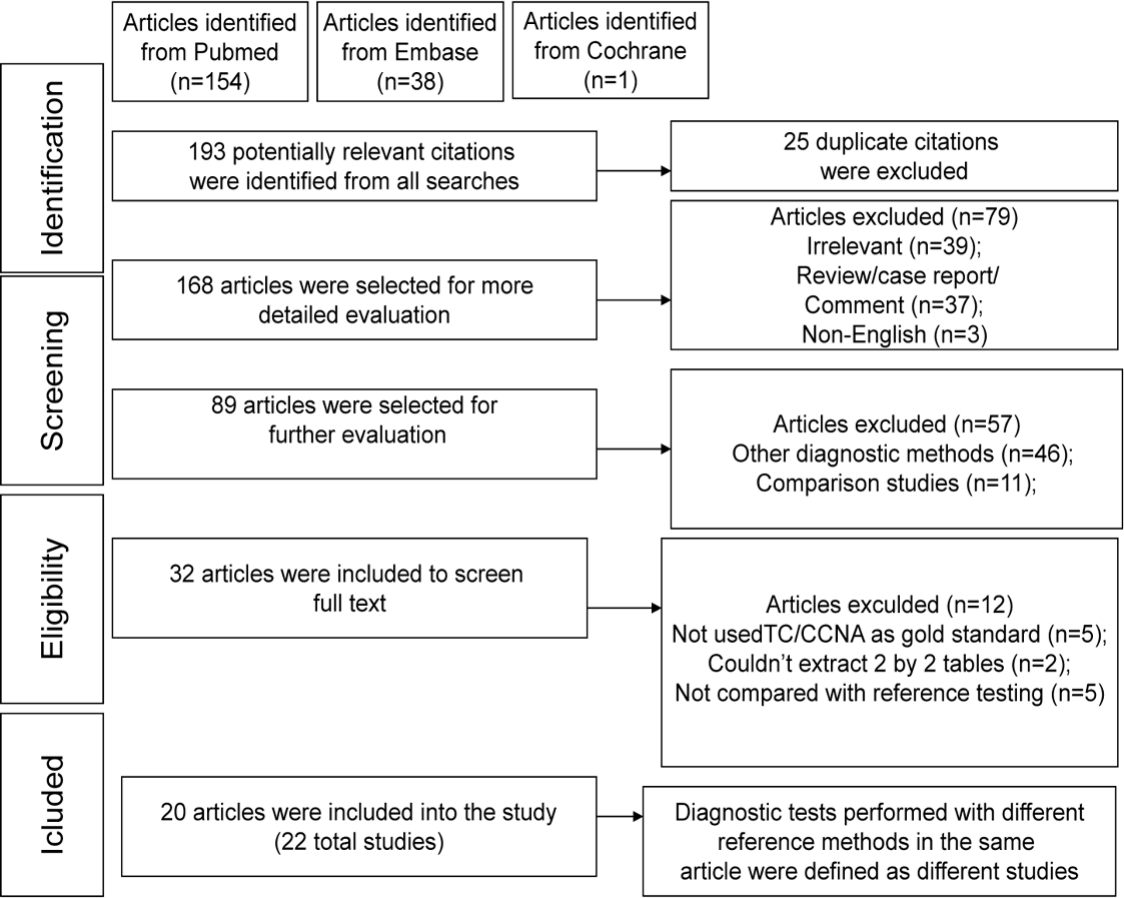
Flow chart of study selection.

A flow chart of the study selection process is shown in Fig 1. A total of 193 potentially relevant citations were identified from all searches. Finally, according to the inclusion and exclusion criteria, 20 eligible articles fulfilled the inclusion criteria and were included in the meta-analysis. Because diagnostic tests performed with different reference methods occurred in the same article, 22 independent studies (including 9352 samples) were defined in the meta-analysis. Table 1 shows the characteristics of these included studies [16–35]. The prevalence of CDI across all studies ranged from 10% to 47.9%. Two studies used CCNA as a reference test [16,19].In one study, the investigators reported the diagnostic accuracy separately for both the reference standards [19]. In another study, the investigators reported the diagnostic accuracy separately for TC and Enriched TC [17]. Most of the studies were prospective in design.

**Table 1:** Summary of the included studies.

First author	Year	Country	Sample size	Reference test	TP ^1)^	FP^2)^	FN^3)^	TN^4)^	Calculated prevalence(%)
Huang [16]	2009	USA	220	CCNA^5)^	34	13	1	172	15.9
Tenover-1[17]	2010	Canada & USA	2296	TC^6)^	245	188	3	1860	10.8
Tenover-2[17]	2010	Canada & USA	2296	Enriched TC	316	117	22	1841	14.7
Novak-Weekley[18]	2010	USA	428	Enriched TC	68	13	4	343	16.8
Swindells-1[19]	2010	UK	150	CCNA	15	4	0	131	10
Swindells-2[19]	2010	UK	150	TC	19	1	0	130	12.7
Goldenberg[20]	2010	UK	224	TC	57	6	0	161	25.4
Dubberke [21]	2011	USA	150	TC	44	7	0	99	29.3
Zidaric [22]	2011	Slovenia	178	TC	27	4	1	146	15.7
Buchan [23]	2012	USA	275	TC	58	18	0	199	21.1
Viala [24]	2012	France	94	TC	44	1	1	48	47.9
Shin [25]	2012	Korea	248	TC	49	10	0	189	19.6
Dalpke [26]	2013	Germany	448	TC	72	8	2	366	16.5
Eigner [27]	2014	Germany	245	TC	74	8	2	161	31
Gilbreath [28]	2014	USA	190	TC	23	2	0	165	12.1
Jensen [29]	2015	Denmark	299	TC	38	20	0	241	16.6
Jazmati [30]	2015	Germany	199	Enriched TC	28	17	0	154	14.1
Yoo [31]	2015	Korea	254	TC	72	2	15	165	34.1
Moon [32]	2016	Korea	258	TC	52	11	3	192	21.3
Moon [33]	2016	Korea	270	TC	52	11	3	204	20.4
Shin [34]	2016	Korea	339	TC	78	18	9	234	25.7
Rajabally [35]	2016	South Africa	141	TC	27	3	3	108	21.3

^1)^*TP*:true positives

^2)^*FP*:false positives

^3)^*FN*:false negatives

^4)^*TN*: true negatives

^5)^*CCNA*: culture cytotoxicity neutralization assay

^6)^*TC*: toxigenic culture

### Quality assessment

**Fig 2:**
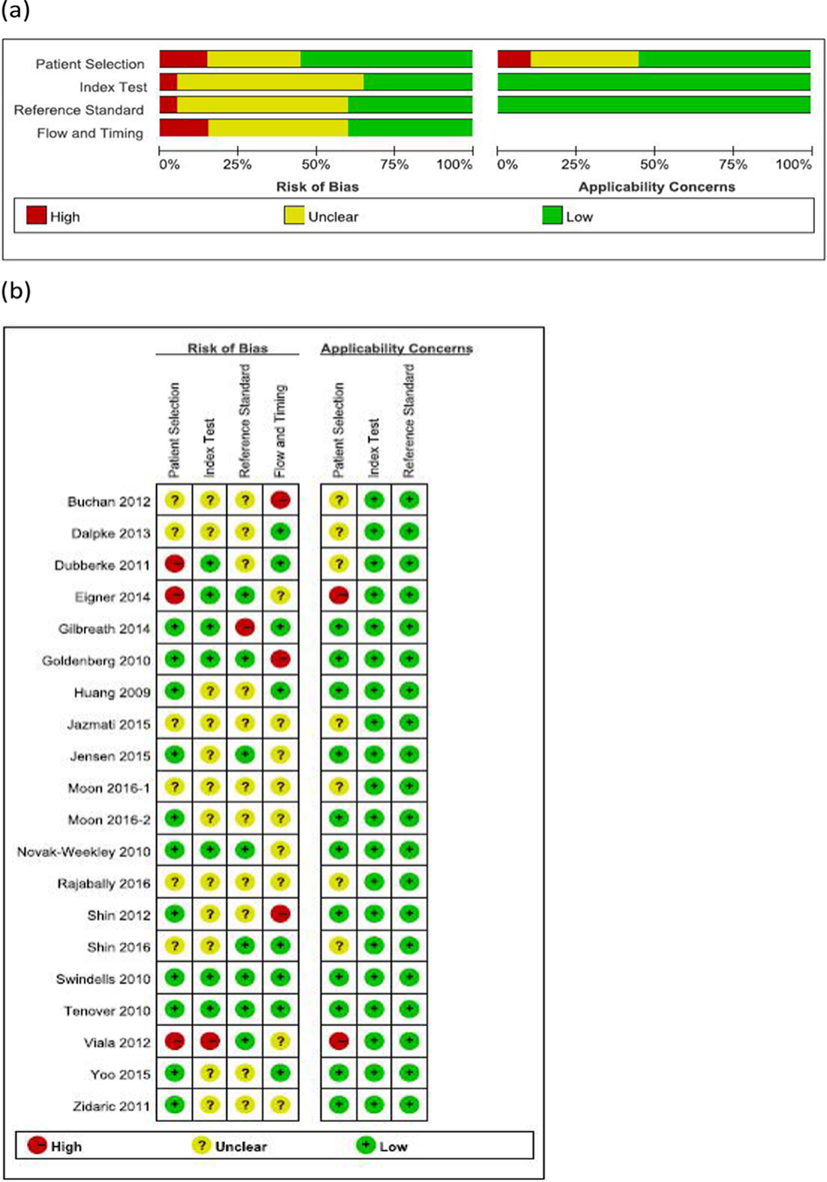
Quality assessment of included studies.

A quality assessment of all of the included articles is illustrated in Fig 2. In conclusion, patient selection was the most high-risk or unclear risk bias and high risk applicability concerns. More than half of the included articles were at either high risk or unclear risk bias in “patient selection” and “flow and timing” domains of QUARDAS-2 due to lack of detail regarding timing, inconsecutive, or nonrandom patient selection and blinding. A total of 9 (45%) articles were at low risk, 7 articles (35%) were of unclear risk, and 4 articles (20%) were at high risk for patient selection bias. A total of 12 articles (60%) were at high risk for flow and timing bias, because of the fact that not all selected patients were included in the diagnostic analysis. Most of the articles were at either low or unclear risk for index test and reference standard bias. Regarding applicability, half of the articles were at high risk for patient selection; however, all selected articles (n = 20, 100%) were at low risk of index test and the reference standard. In conclusion, patient selection was the most high-risk or unclear risk bias and high risk applicability concerns.

### Diagnostic accuracy

**Fig 3:**
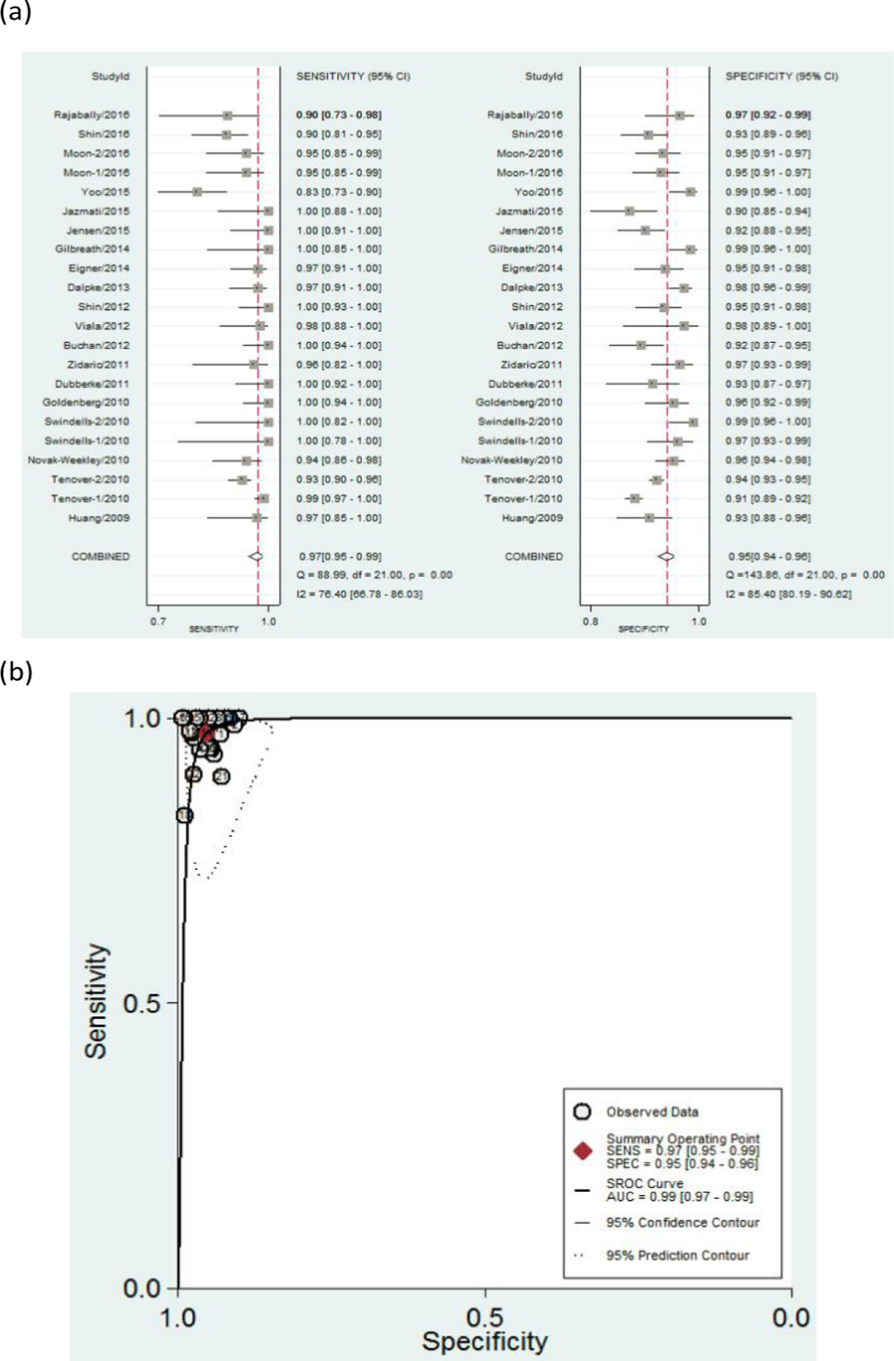
Forest plots of the pooled sensitivity and specificity and SROC curve of Xpert CD for detection of CDI. (a) Forest plots of the pooled sensitivity and specificity. Each solid square represents an individual study. Error bars represent 95% CI. Diamond indicates the pooled sensitivity and specificity for all of the studies. (b). SROC curve.

Results are given as values (95% CI). Using a random-effects model, the results were as follows: sensitivity 0.97(0.95–0.99), *I*^*2*^ = 76.4%; specificity 0.95(0.94–0.96), *I*^*2*^ = 85.4% (Fig 3); PLR 21.41(16.66–27.52), *I*^*2*^ = 78.9%; NLR 0.03 (0.02–0.05), *I*^*2*^ = 72.55%; DOR 762.13(401.82–1445.52), *I*^*2*^ = 100%; and AUC 0.99 (0.97–0.99) (Fig 3). The results indicated a good level of overall accuracy.

**Fig 4:**
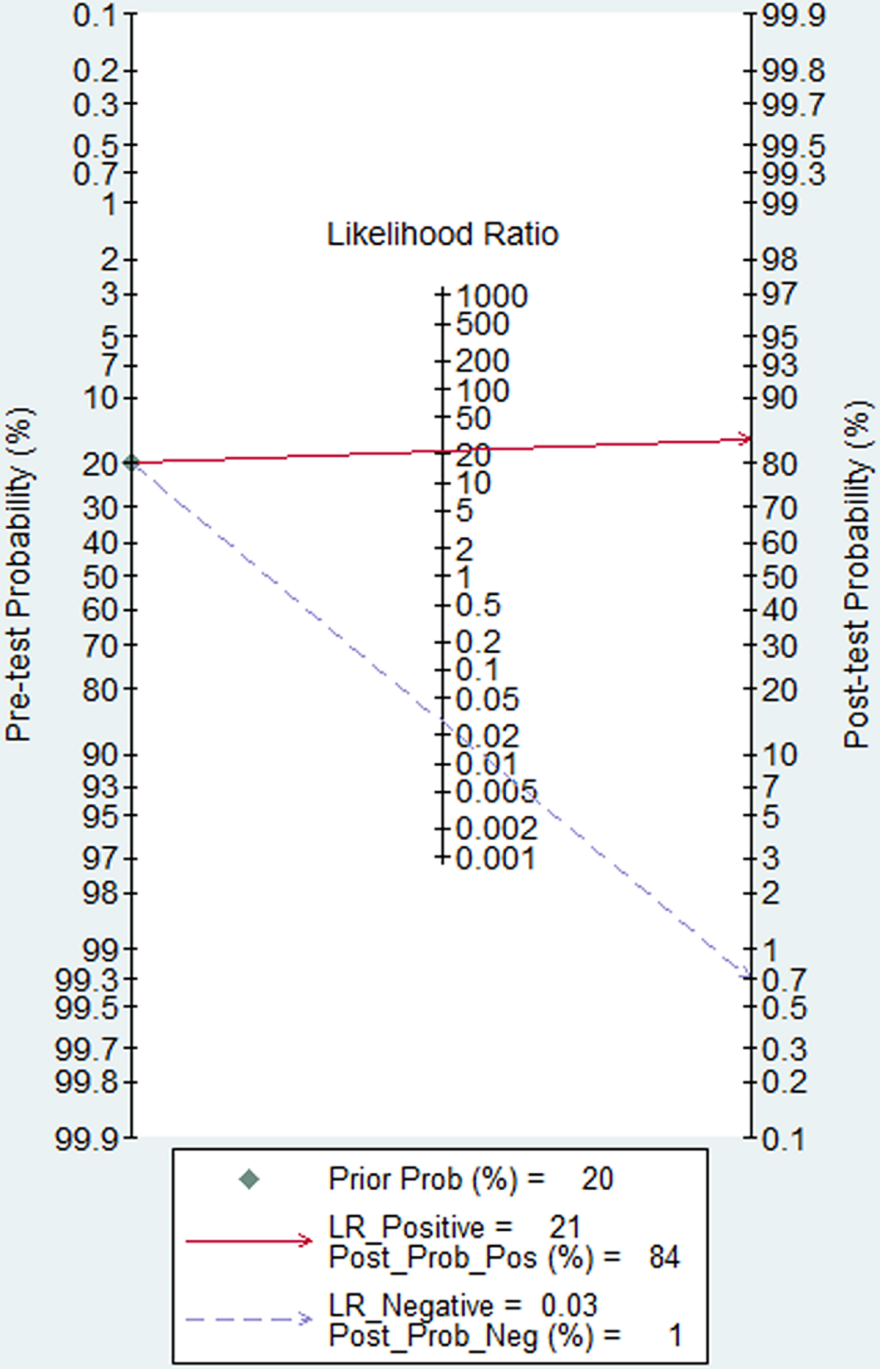
Fagan nomogram of Xpert CD assay for diagnosis of CDI.

The relationship between pretest probability and posttest probability was depicted by visual Fagan's nomogram. For patients with a pretest probability of 20%, the posttest probability of positive results was 84%, and posttest probability of negative results was 1% (Fig 4).

#### Heterogeneity

There was substantial heterogeneity for all the statistical measures. The heterogeneity test results of sensitivity and specificity are illustrated in the forest plots (Fig 3).The Spearman correlation coefficient between the logit of sensitivity and logit of 1-specificity was used to assess the threshold/cut-off effect. The Spearman correlation coefficient (*p* value) in diagnostic of CDI was 0.270 (*p* = 0.201). This indicated that the heterogeneity might not be due to threshold/cut-off effect. To assess for causes of variations other than threshold, we performed subgroup analyses in terms of CDI prevalence and sample size.

#### Subgroup analyses

At a prevalence of <15% (6 studies, 5281samples), the sensitivity was 0.99 (0.89–1.00), *I*^*2*^ = 86.4%; specificity, 0.96 (0.92–0.98), *I*^*2*^ = 97.5%; PLR 25.07 (11.68–53.79), *I*^*2*^ = 96.4%; NLR 0.03 (0.02–0.05), *I*^*2*^ = 85.25%; DOR 2672.14 (141.68–50398.07), *I*^*2*^ = 100%; and AUC 0.99 (0.97–0.99).

At a prevalence of >15% (16 studies, 4071samples), the sensitivity was 0.97 (0.94–0.99), *I*^*2*^ = 71.56%; specificity, 0.95 (0.94–0.96), *I*^*2*^ = 54.45%; PLR 21.22 (16.73–26.91), *I*^*2*^ = 25.39%; NLR 0.03 (0.02–0.06), *I*^*2*^ = 66.83%; DOR 681.72 (344.60–1348.67), *I*^*2*^ = 99.82%; and AUC 0.98 (0.97–0.99).

The median sample size was 246. In studies with a sample size <246 (11 studies, 1941 samples), the sensitivity was 0.98 (0.95–0.99), *I*^*2*^ = 39.1%; specificity 0.96 (0.94–0.98), *I*^*2*^ = 70.52%; PLR 27.05 (17.65–41.46), *I*^*2*^ = 51.46%; NLR 0.02 (0.01–0.05), *I*^*2*^ = 15.31%; DOR 1420.95 (495.97–4071.02), *I*^*2*^ = 73.31%; and AUC 1.00 (0.98–1.00).

In studies with a sample size >246 (11 studies, 7411 samples), the sensitivity was 0.96(0.93–0.98), *I*^*2*^ = 81.25%; specificity 0.95 (0.95–0.96), *I*^*2*^ = 84.85%; PLR 18.21 (13.81–24.01), *I*^*2*^ = 72.93%; NLR 0.04 (0.02–0.08), *I*^*2*^ = 78.42%; DOR 454.58 (239.02–864.57), *I*^*2*^ = 98.09%; and AUC 0.98(0.97–0.99).

**Fig 5:**
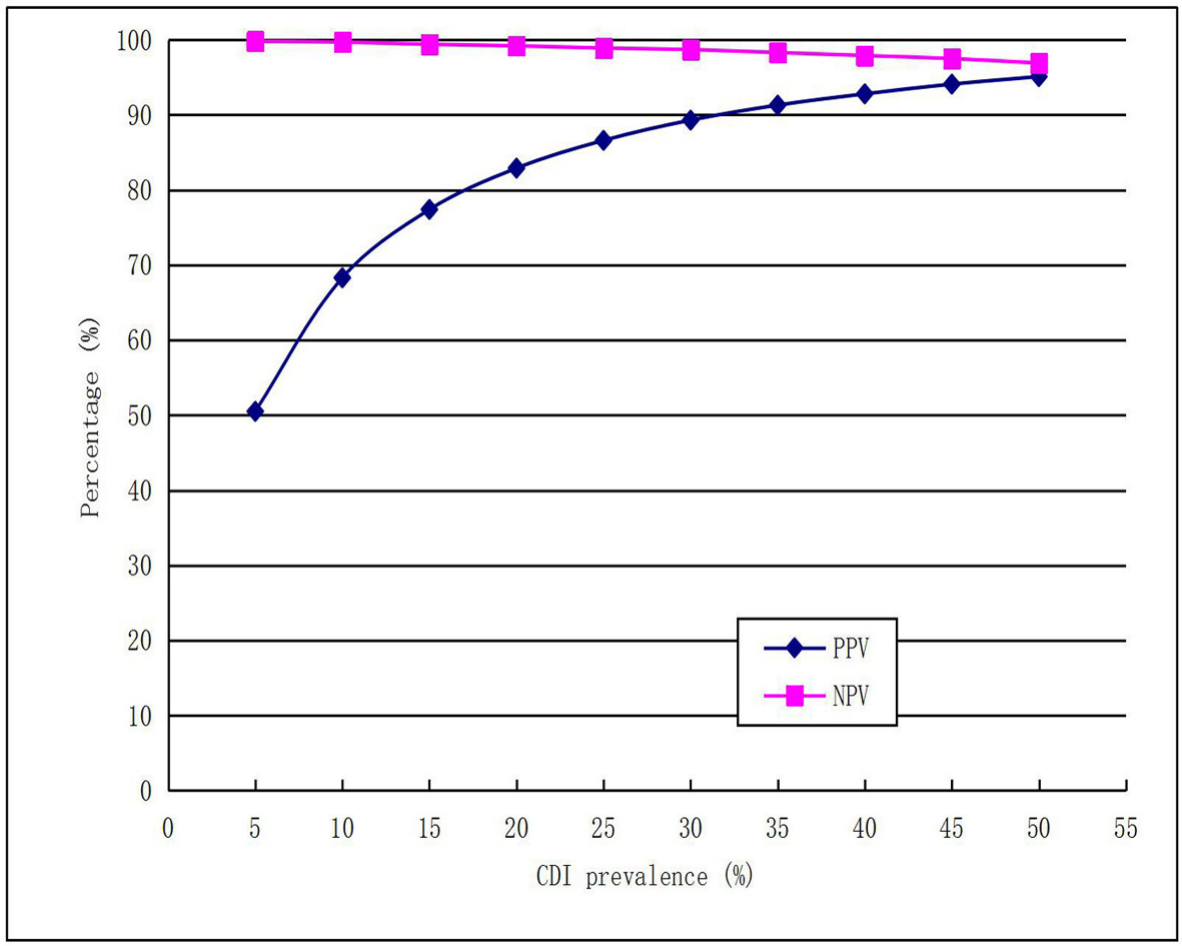
Theoretical values of PPV and NPV for increasing CDI prevalence calculated using pooled sensitivity (97%) and specificity (95%).

The theoretical values of PPV and NPV were calculated using the pooled sensitivity (0.97) and specificity (0.95) values and plotted against increasing CDI prevalence. The PPV performance is variable and correlated positively with increasing CDI prevalence, whereas NPV remained almost quite high (Fig 5).

## Discussion

In recent years, NAATs for the direct detection of *C*. *difficile* toxin genes in stool samples have garnered strong research interest worldwide and are a highly sensitive alternative to the EIAs, the time-consuming TC and CCNA. To that end, we focused on the Xpert CD assay which has been cleared by FDA to rapidly diagnose patients with CDI. The Xpert CD assay is now implemented in many countries due to its shorter turnaround time, thus a more effective procedure. The most significant advantage of the Xpert CD assay is its rapidity and simplicity. As described previously [9,28], besides detecting toxigenic *C*. *difficile*, the Xpert CD assay reports presumptive identification of ‘hypervirulent’ 027/NAP1/BI (positive for *cdt* and *tcdC* Δ117). The 14-day mortality was very high for 027 /NAP1/BI (20%), compared to an overall mortality of 13% (p < 0.0001) [36]. A previously published study reported that the agreement between the Xpert CD assay and PCR-ribotyping was 93% [37]. And another study found “very good” agreement at 97.9% between this assay and multilocus sequence typing (MLST) for identification of *C*. *difficile* NAP1 [38]. Recently, studies focusing on the diagnostic accuracy of the Xpert CD assay were conducted in many settings, but with inconsistent results. To provide much more evidence-based results for utility of this assay in routine testing, we conducted this meta-analysis to evaluate the diagnostic accuracy of the Xpert CD assay for direct detection of CDI compared with conventional reference tests.

In the literature there are two meta-analyses in which the Xpert CD assay has been assessed [39,40]. The first analysis, performed in 2012, was limited by only including four studies that could not fully assess the clinical application of this assay [39]. The second analysis, as a part of a review published in 2013, focused on three popular commercial NAATs, including the Xpert CD assay, only evaluated the assay compared to TC and only reported pooled sensitivity [40]. As we know, to evaluate the diagnostic accuracy of this assay, other measures such as specificity, DOR, PLR and NLR also should be reported. Our meta-analysis identified several additional studies since the publication of the two reviews. To the best of our knowledge, the present meta-analysis, with 22 studies included, is the first study that has comprehensively evaluated the overall diagnostic accuracy of the Xpert CD assay in detecting toxigenic CDI. In our meta-analysis, the Xpert CD assay showed high pooled sensitivity (97%) and specificity (95%) for detection of CDI, with lower specificity than sensitivity which was one of the common characteristics of NAATs. While NAATs have highly sensitivity, there are some important issues that have been raised regarding their practical application. The main issue regarding the clinical utility of NAATs, especially the PPV and specificity, is linked to the detection of genes versus actual toxins. Someone concerned that NAATs can be positive in both disease states and colonization [9]. Detection of toxin genes does not necessarily correlate with expression of toxin, nor disease. Another limitation of the NAATs is that nearly all assays focus on *tcdB* detection, but if a strain did not produce toxin B or was *tcdB*-variant, it would led to a false-negative result. Some researches have reported that inappropriate test ordering can also impact the clinical specificity of tests [21].

Given a pretest probability of 20%, the posttest probability for a positive test results was 84%, and posttest probability of negative test result was 1%. The PPV performance correlated positively with increasing CDI prevalence.This meta-analysis showed that Xpert CD assay had high mean DOR and large AUC values, indicating a high value of overall accuracy for the detection of CDI. A quite high PLR and a very low NLR for the detection CDI in our study indicated an excellent ability to both confirm and exclude CDI. The better diagnostic accuracy found in our study may provide more powerful evidence for routine clinical application of Xpert CD assay.

We found significant heterogeneity for diagnostic parameters among the studies analyzed. The Spearman correlation coefficient between the logit of sensitivity and logit of 1-specificity was not significant, indicating that the heterogeneity was not caused by threshold/cut-off effect. Thus, subgroup analyses based on CDI prevalence and sample size were performed to test for causes of variations other than threshold effect. There were no significant heterogeneity for PLR (*I*^*2*^ = 25.39%, *p* = 0.01) when CDI prevalence of studies greater than 15% were pooled and that for sensitivity (*I*^*2*^ = 39.1%, *p* = 0.09) and NLR (*I*^*2*^ = 15.31%, *p* = 0.30) when sample size of studies less than median sample size were pooled. The results suggested that the CDI prevalence and sample size could partly explain the heterogeneity. Even so, the considerable heterogeneity in the results remained unexplained, which may be caused by the various baseline criteria for accepting stool samples for testing.

Our meta-analysis had several methodological strengths, such as standard protocol and rigorous statistical methods. However, our meta-analysis also had several limitations. First, the studies included in this analysis have various baseline criteria for accepting stool samples, different patient populations and institutional characteristics. Most studies did not specify the exact criteria used to submit patient stool samples to the laboratory for testing, which made it difficult to avoid detecting asymptomatic carriers. Second, the present authors only included studies published in English, and some studies missing data to calculate sensitivity and specificity were excluded since the authors could not be contacted. Moreover, we did not show the detailed data about analyzing studies that used TC or CCNA or enriched TC as the reference standard separately, because of only 2 studies used CCNA and 3 studies used enriched TC as reference tests. It did not reduce potential heterogeneity when studies with TC as reference method were pooled (data not shown).

In general, although the Xpert CD assay showed good accuracy for CDI detection in this meta-analysis, some important issues remain to be addressed. Like the problems about NAATs we mentioned above, one of the more important questions concern the clinical utility of this assay is that it specifically detects the *tcdB* gene encoding the toxin and not an actual toxin. Therefore, asymptomatic carriers can be misdiagnosed as disease state if inappropriate testing is performed. The asymptomatic colonization rate is about 2% in healthy adults and can be as high as 51% for residents of long term care facilities [41]. To avoid over diagnosis and overtreatment of toxigenic CDI by using the Xpert CD assay, it must be strictly limited to diarrheal stool specimen in patients without laxatives. The clinician should be mindful of the limits of this rapid molecular assay and clinical assessment is necessary to detect true infection. Also, the assay can remain positive for weeks after the resolution of clinical symptoms and should not be repeated for monitoring treatment.

Another important issue of cost-effectiveness of the molecular methods, including the Xpert CD assay, has been raised by laboratorians and administrators. The molecular assays cost up to 2- to 3-fold more than EIAs. To lessen the cost of NAATs, a two-step algorithm based on glutamate dehydrogenase (GDH) detection and NAAT in case of a positive result constitute an excellent alternative to the exclusive use of NAATs. Culbreath *et al*. reported that this algorithm was 56% cheaper than applying the Xpert CD systematically in their institution (US$70,633 *vs*. US$159,877 per 1000 tests) [42]. Given the rapidity and simplicity of the Xpert CD assay that are helpful for timely implementation of appropriate therapy and contact precaution, this two-step algorithms may not offer an advantage but may be used for cost savings. On the other hand, the higher cost of NAATs may be counterbalanced by a decrease in healthcare-associated infections costs. Babady *et al*. reported that performing the Xpert CD test alone was less expensive than two-step algorithms when labor costs (accessioning, test performance, and reporting of results) were considered [43]. In the future, it is needed to assess the overall cost-effectiveness of Xpert CD for the diagnosis of *C*. *difficile* disease, including comprehensive laboratory costs and overall hospital costs.

In conclusion, the present meta-analysis showed that the Xpert CD assay had good accuracy for detecting CDI, suggesting that it has good utility as a rapid screening molecular tool. In the future, studies are needed to focus on the prediction of the disease severity. While several biomarkers that correlates with active CDI have been evaluated (such as fecal lactoferrin, calprotectin, interleukin-8), combining the Xpert CD assay with biomarkers to diagnosis active CDI will likely be an area of investigation in the coming years. This assay will probably be considered as one of the standard diagnostic tests for CDI, either as a standalone test or included in a multistep algorithm.

## Supporting information

S1 File PRISMA checklist. (DOC)

## References

[1] Bartlett JG. Antibiotic-associated diarrhea. Clin Infect Dis. 1992; 15(4):573–581. 1420669 10.1093/clind/15.4.573

[2] Jones AM, Kuijper EJ, Wilcox MH. Clostridium difficile: a European perspective. J Infect. 2013; 66(2), 115–128. doi: 10.1016/j.jinf.2012.10.019 23103666 10.1016/j.jinf.2012.10.019

[3] Zimlichman E, Henderson D, Tamir O, Franz C, Song P, Yamin CK, et al Health care-associated infections: a meta-analysis of costs and financial impact on the US health care system. JAMA Intern Med 2013; 173:2039–46. doi: 10.1001/jamainternmed.2013.9763 23999949 10.1001/jamainternmed.2013.9763

[4] Kuehne SA, Cartman ST, Heap JT, Heap JT, Kelly ML, Cockayne A, et al The role of toxin A and toxin B in Clostridium difficileinfection. Nature 2010; 467(7316), 711–3. doi: 10.1038/nature09397 20844489 10.1038/nature09397

[5] Eckert C, Coignard B, Hebert M, Tarnaud C, Tessier C, Lemire A, et al Clinical and microbiological features of Clostridium difficile infections in France: the ICD-RAISIN 2009 national survey. Med Mal Infect 2013; 43(2), 67–74. doi: 10.1016/j.medmal.2013.01.004 23498135 10.1016/j.medmal.2013.01.004

[6] Loo VG, Poirier L, Miller MA, Oughton M, Libman MD, Michaud S, et al A predominantly clonal multi-institutional outbreak of Clostridium difficile-associated diarrhea with high morbidity and mortality. N Engl J Med 2005; 353: 2442–9. doi: 10.1056/NEJMoa051639 16322602 10.1056/NEJMoa051639

[7] Barbut F, Surgers L, Eckert C, Visseaux B, Cuingnet M, Mesquita C et al Does a rapid diagnosis of Clostridium difficile infection impact on quality of patient management? Clin Microbiol Infect. 2014; 20:136–144. doi: 10.1111/1469-0691.12221 23565919 10.1111/1469-0691.12221

[8] Planche T, Aghaizu A, Holliman R, Riley P, Poloniecki J, Breathnach A, et al Diagnosis of Clostridium difficile infection by toxin detection kits: a systematic review. Lancet Infect Dis 2008; 8:777–84. doi: 10.1016/S1473-3099(08)70233-0 18977696 10.1016/S1473-3099(08)70233-0

[9] Burnham CA, Carroll KC. Diagnosis of Clostridium difficile Infection: an Ongoing Conundrum for Clinicians and for Clinical Laboratories. Clin Microbiol Rev. 2013; 26(3): 604–630. doi: 10.1128/CMR.00016-13 23824374 10.1128/CMR.00016-13 PMC3719497

[10] American Society for Microbiology. A practical guidance document for the laboratory detection of toxigenic Clostridium difficile [update of September 9, 2010, version]. September 21, 2010.

[11] Rupnik M. Heterogeneity of large clostridial toxins: importance of Clostridium difficile toxinotypes. FEMS Microbiol Rev. 2008; 32:541–55. doi: 10.1111/j.1574-6976.2008.00110.x 18397287 10.1111/j.1574-6976.2008.00110.x

[12] Whiting PF, Rutjes AW, Westwood ME. QUADAS-2: a revised tool for the quality assessment of diagnostic accuracy studies. Ann Intern Med. 2011; 155(8):529–36. doi: 10.7326/0003-4819-155-8-201110180-00009 22007046 10.7326/0003-4819-155-8-201110180-00009

[13] Whiting PF, Rutjes AW, Westwood ME.QUADAS-2: a revised tool for the quality assessment of diagnostic accuracy studies. Ann Intern Med. 2011; 155(8):529–536. doi: 10.7326/0003-4819-155-8-201110180-00009 22007046 10.7326/0003-4819-155-8-201110180-00009

[14] Bai YY, Wang YL, Shao CH, Hao YY, Jin Y. Genotype MTBDR plus assay for rapid detection of multidrug resistance in Mycobacterium tuberculosis: a meta analysis. 10.1371/journal.pone.0150321 PMC4774872 26934724

[15] Lloyd A, Pasupuleti V, Thota P, Pant C, Rolston DD, Hernandez AV, et al Accuracy of loop-mediated isothermal amplification for the diagnosis of Clostridium difficile infection: a systematic review. Diagn Microbiol Infect Dis. 2015; 82(1):4–10. doi: 10.1016/j.diagmicrobio.2015.02.007 25752201 10.1016/j.diagmicrobio.2015.02.007

[16] Huang H, Weintraub A, Fang H, Nord CE. Comparison of a commercial multiplex real-time PCR to the cell cytotoxicity neutralization assay for diagnosis of clostridium difficile infections.J Clin Microbiol 2009;47(11):3729–31. doi: 10.1128/JCM.01280-09 19741082 10.1128/JCM.01280-09 PMC2772590

[17] Tenover FC, Novak-Weekley S, Woods CW, Peterson LR, Davis T, Schreckenberger P, et al Impact of strain type on detection of toxigenic Clostridium difficile: comparison of molecular diagnostic and enzyme immunoassay approaches. J Clin Microbiol 2010; 48(10): 3719–24. doi: 10.1128/JCM.00427-10 20702676 10.1128/JCM.00427-10 PMC2953097

[18] Novak-Weekley SM, Marlowe EM, Miller JM, Cumpio J, Nomura JH, Vance PH, et al Clostridium difficile testing in the clinical laboratory by use of multiple testing algorithms. J Clin Microbiol 2010; 48(3):889–93. doi: 10.1128/JCM.01801-09 20071552 10.1128/JCM.01801-09 PMC2832460

[19] Swindells J, Brenwald N, Reading N, et al Evaluation of diagnostic tests for Clostridium difficile infection. J Clin Microbiol 2010; 48(2):606–8. doi: 10.1128/JCM.01579-09 20032256 10.1128/JCM.01579-09 PMC2815642

[20] Goldenberg SD, Dieringer T, French GL. Detection of toxigenic Clostridium difficile in diarrheal stools by rapid real-time polymerase chain reaction. Diagn Microbiol Infect Dis 2010; 67(3): 304–7. doi: 10.1016/j.diagmicrobio.2010.02.019 20542211 10.1016/j.diagmicrobio.2010.02.019

[21] Dubberke ER, Han Z, Bobo L, Hink T, Lawrence B, Copper S, et al Impact of clinical symptoms on interpretation of diagnostic assays for Clostridium difficile infections. J Clin Microbiol 2011; 49(8): 2887–93. doi: 10.1128/JCM.00891-11 21697328 10.1128/JCM.00891-11 PMC3147743

[22] Zidarič V, Kevorkijan BK, Oresic N, Janezic S, Rupnik M. Comparison of two commercial molecular tests for the detection of Clostridium difficile in the routine diagnostic laboratory. J Med Microbiol 2011; 60(Pt 8): 1131–6. doi: 10.1099/jmm.0.030163-0 21372187 10.1099/jmm.0.030163-0

[23] Buchan BW, Mackey TL, Daly JA, Alger G, Denys GA, Peterson LR, et al Multicenter clinical evaluation of the portrait toxigenic C. difficile assay for detection of toxigenic Clostridium difficile strains in clinical stool specimens. J Clin Microbiol 2012; 50(12): 3932–6. doi: 10.1128/JCM.02083-12 23015667 10.1128/JCM.02083-12 PMC3502949

[24] Viala C, Le Monnier A, Maataoui N, Rousseau C, Collignon A, Poilane I. Comparison of commercial molecular assays for toxigenic Clostridium difficile detection in stools: BD GeneOhm Cdiff, XPert C. difficile and illumigene C. difficile. J Microbiol Methods 2012; 90(2): 83–5. doi: 10.1016/j.mimet.2012.04.017 22565213 10.1016/j.mimet.2012.04.017

[25] Shin S, Kim M, Kim M, Lim H, Kim H, Lee K, et al Evaluation of the Xpert Clostridium difficile assay for the diagnosis of Clostridium difficile infection. Ann Lab Med 2012; 32(5): 355–8. doi: 10.3343/alm.2012.32.5.355 22950071 10.3343/alm.2012.32.5.355 PMC3427823

[26] Dalpke AH, Hofko M, Zorn M, Zimmermann S. Evaluation of the fully automated BD MAX Cdiff and Xpert C. difficile assays for direct detection of Clostridium difficile in stool specimens. J Clin Microbiol 2013; 51(6): 1906–8. doi: 10.1128/JCM.00344-13 23515539 10.1128/JCM.00344-13 PMC3716071

[27] Eigner U, Fenner I, Veldenzer A, Schwarz R, Oberdorfer K, Holfelder M, et al Evaluation of six PCR assays in combination with patient related data for the diagnosis of Clostridium difficile-associated infections. Clin Lab 2014; 60(8): 1343–50. 25185420 10.7754/clin.lab.2013.130735

[28] Gilbreath JJ, Verma P, Abbott AN, Butler-Wu SM. Comparison of the Verigene Clostridium difficile, Simplexa C. difficile Universal Direct, BD MAX Cdiff, and Xpert C. difficile assays for the detection of toxigenic C. difficile. Diagn Microbiol Infect Dis 2014; 80(1): 13–8. doi: 10.1016/j.diagmicrobio.2014.06.001 25027069 10.1016/j.diagmicrobio.2014.06.001

[29] Jensen MB, Olsen KE, Nielsen XC, Hoegh AM, Dessau RB, Atlung T, et al Diagnosis of Clostridium difficile: real-time PCR detection of toxin genes in faecal samples is more sensitive compared to toxigenic culture. Eur J Clin Microbiol Infect Dis 2015; 34(4): 727–36. doi: 10.1007/s10096-014-2284-7 25421216 10.1007/s10096-014-2284-7

[30] Jazmati N, Wiegel P, Ličanin B, Plum G. Evaluation of the Qiagen artus C. difficile QS-RGQ Kit for Detection of Clostridium difficile Toxins A and B in Clinical Stool Specimens. J Clin Microbiol 2015; 53(6): 1942–4. doi: 10.1128/JCM.00613-15 25809977 10.1128/JCM.00613-15 PMC4432059

[31] Yoo J, Lee H, Park KG, Lee GD, Park YG, Park YJ. Evaluation of 3 automated real-time PCR (Xpert C. difficile assay, BD MAX Cdiff, and IMDx C. difficile for Abbott m2000 assay) for detecting Clostridium difficile toxin gene compared to toxigenic culture in stool specimens.Diagn Microbiol Infect Dis 2015; 83(1): 7–10. doi: 10.1016/j.diagmicrobio.2015.05.005 26081240 10.1016/j.diagmicrobio.2015.05.005

[32] Moon HW, Kim HN, Kim JY, Hur M, Kim H, Yun YM. Performance of the artus C. difficile QS-RGQ Kit for the detection of toxigenic Clostridium difficile. Clin Biochem 2016; 22. pii: S0009-9120(16)30205-3. 10.1016/j.clinbiochem.2016.08.013 27556286

[33] Moon HW, Kim HN, Hur M, Shim HS, Kim H, Yun YM, et al Comparison of Diagnostic Algorithms for Detecting Toxigenic Clostridium difficile in Routine Practice at a Tertiary Referral Hospital in Korea. PLoS One 2016; 11(8):e0161139 doi: 10.1371/journal.pone.0161139 27532104 10.1371/journal.pone.0161139 PMC4988646

[34] Shin BM, Yoo SM, Shin WC. Evaluation of Xpert C. difficile, BD MAX Cdiff, IMDx C. difficile for Abbott m2000, and Illumigene C. difficile Assays for Direct Detection of Toxigenic Clostridium difficile in Stool Specimens. Ann Lab Med 2016; 36(2): 131–7. doi: 10.3343/alm.2016.36.2.131 26709260 10.3343/alm.2016.36.2.131 PMC4713846

[35] Rajabally N, Kullin B, Ebrahim K, Brock T, Weintraub A, Whitelaw A, et al A comparison of Clostridium difficile diagnostic methods for identification of local strains in a South African centre. J Med Microbiol 2016 2 9. doi: doi: 10.1099/jmm.0.000231. [Epub ahead of print] 26860329 10.1099/jmm.0.000231

[36] Walker AS, Eyre DW, Wyllie DH, Dingle KE, Griffiths D, Shine B, et al Relationship between bacterial strain type, host biomarkers, and mortality in Clostridium difficile infection. Clin Infect Dis 2013; 56(11): 1589–1600. doi: 10.1093/cid/cit127 23463640 10.1093/cid/cit127 PMC3641870

[37] Babady NE, Stiles J, Ruggiero P, Khosa P, Huang D, Shuptar S, et al Evaluation of the Cepheid Xpert Clostridium difficile Epi assay for diagnosis of Clostridium difficile infection and typing of the NAP1 strain at a cancer hospital. J Clin Microbiol 2010; 48: 4519–24. doi: 10.1128/JCM.01648-10 20943860 10.1128/JCM.01648-10 PMC3008447

[38] McMillen T, Kamboj M, Babady NE. Comparison of Multilocus Sequence Typing and the Xpert C. difficile/Epi Assay for Identification of Clostridium difficile 027/NAP1/BI. J Clin Microbiol. 2016; 54(3):775–8. doi: 10.1128/JCM.03075-15 26699700 10.1128/JCM.03075-15 PMC4767941

[39] O'Horo JC, Jones A, Sternke M, Harper C, Safdar N. Molecular techniques for diagnosis of Clostridium difficile infection: systematic review and meta-analysis. Mayo Clin Proc. 2012; 87(7): 643–51. doi: 10.1016/j.mayocp.2012.02.024 22766084 10.1016/j.mayocp.2012.02.024 PMC3538482

[40] Le Guern R, Herwegh S, Courcol R, Wallet F. Molecular methods in the diagnosis of Clostridium difficile infections: an update. Expert Rev Mol Diagn. 2013; 13(7): 681–92. doi: 10.1586/14737159.2013.829705 24063396 10.1586/14737159.2013.829705

[41] Aronsson B, Mollby R, Nord CE. Antimicrobial agents and Clostridium difficile in acute enteric disease: epidemiological data from Sweden, 1980–1982. J Infect Dis. 1985; 151(3): 476–481. 3973405 10.1093/infdis/151.3.476

[42] Culbreath K, Ager E, Nemeyer RJ, Kerr A, Gilligan PH. Evolution of Testing Algorithms at a University Hospital for Detection of Clostridium difficile Infections. J Clin Microbiol 2012; 50(9): 3073–3076. doi: 10.1128/JCM.00992-12 22718938 10.1128/JCM.00992-12 PMC3421798

[43] Chapin K. Discrepancies in testing recommendations for Clostridium difficile infection: updated review favors amplification test systems. Expert Rev Mol Diagn. 2012; 12(3), 223–226. doi: 10.1586/erm.12.13 22468811 10.1586/erm.12.13

